# BNIP3-dependent mitophagy safeguards ESC genomic integrity via preventing oxidative stress-induced DNA damage and protecting homologous recombination

**DOI:** 10.1038/s41419-022-05413-4

**Published:** 2022-11-19

**Authors:** Qian Zhao, Kun Liu, Lin Zhang, Zheng Li, Liang Wang, Jiani Cao, Youqing Xu, Aihua Zheng, Quan Chen, Tongbiao Zhao

**Affiliations:** 1 State Key Laboratory of Stem Cell and Reproductive Biology, Institute for Stem Cell and Regeneration, Institute of Zoology, Chinese Academy of Sciences Beijing, Beijing, 100101 China; 2 Beijing Institute for Stem Cell and Regenerative Medicine, Beijing, 100101 China; 3 University of Chinese Academy of Sciences, Beijing, 100049 China; 4 Department of Gastroenterology, Beijing Tiantan Hospital, Capital Medical University, Beijing, 100070 China; 5 College of Life Sciences, Nankai University, Tianjin, 300073 China

**Keywords:** Embryonic stem cells, Mitophagy

## Abstract

Embryonic stem cells (ESCs) have a significantly lower mutation load compared to somatic cells, but the mechanisms that guard genomic integrity in ESCs remain largely unknown. Here we show that BNIP3-dependent mitophagy protects genomic integrity in mouse ESCs. Deletion of *Bnip3* increases cellular reactive oxygen species (ROS) and decreases ATP generation. Increased ROS in *Bnip3*^*−/−*^ ESCs compromised self-renewal and were partially rescued by either NAC treatment or p53 depletion. The decreased cellular ATP in *Bnip3*^*−/−*^ ESCs induced AMPK activation and deteriorated homologous recombination, leading to elevated mutation load during long-term propagation. Whereas activation of AMPK in X-ray-treated *Bnip3*^*+/+*^ ESCs dramatically ascended mutation rates, inactivation of AMPK in *Bnip3*^*−/−*^ ESCs under X-ray stress remarkably decreased the mutation load. In addition, enhancement of BNIP3-dependent mitophagy during reprogramming markedly decreased mutation accumulation in established iPSCs. In conclusion, we demonstrated a novel pathway in which BNIP3-dependent mitophagy safeguards ESC genomic stability, and that could potentially be targeted to improve pluripotent stem cell genomic integrity for regenerative medicine.

## Introduction

Pluripotent stem cells (PSCs), including embryonic stem cells (ESCs) and induced pluripotent stem cells (iPSCs), can undergo unlimited self-renewal and maintain potency to differentiate into every cell type of an organism [1–6]. PSCs can be cultured and propagated indefinitely in vitro with a high proliferation rate, thus potentially providing an unlimited renewable source for various functional cells to serve regenerative medicine. Existing studies have shown that intrinsic characters like DNA-replications stress and environmental culture conditions both endorse PSCs easy to acquire genetic variations, which pose a serious risk for genomic instability [7–11]. Acquired DNA lesions are detrimental to PSC maintenance and function, and also raise safety concerns about the development of PSC-based cellular therapies.

In nature, ESCs, the equivalent to the inner cell mass of developing blastocysts, have a significantly lower mutation load and a higher proliferation rate with a short G1 phase compared to their differentiated progeny cells, suggesting that ESCs have an effective DNA damage response (DDR) to control their pronounced propensity to acquire DNA defects [12–20]. ESCs generate lower amounts of the genotoxic byproducts reactive oxygen species (ROS) and have increased expression of antioxidants compared to somatic cells [19, 21, 22]. In addition, DNA repair efficiency of ESCs in response to DNA damage is enhanced to protect their genomic stability relative to somatic cells. Somatic cells use error-prone non-homologous end joining (NHEJ) to repair most DNA double strand breaks (DSBs), whereas in PSCs error-free homologous recombination (HR) predominates and allows repair of DSBs with increased fidelity [23]. Compared to somatic cells, ESCs have higher levels of ATM/ATR-dependent phosphorylation of H2AX (γH2AX) and chromatin loading of single-stranded DNA (ssDNA)-binding proteins PRA32 and RAD51, which are both important for HR at DNA DSBs and stalled forks, indicating that ESCs could maintain a sensitized DDR state to mitigate DNA damage risks [24–26]. When DNA damage is not repaired, ESCs avoid the propagation of genetic lesions by undergoing either apoptosis or differentiation [18, 27–32]. Despite these multiple reports showing that PSCs have more efficient DNA repair capacity than somatic cells, the mechanisms responsible for these processes are poorly defined.

Mitochondria in human ESCs maintain a high mitochondrial priming state that sensitizes hESCs to DNA-damage-induced apoptosis, and favors the elimination of cells with DNA damage from self-renewing cell pools [27]. This finding indicates that mitochondria are involved in the maintenance of ESC genomic integrity. Mitochondria are highly dynamic, double-membrane organelles that maintain homeostasis through autophagy [33]. In mouse ESCs, dysfunction of autophagy by depletion of ATG3 or ATG5 results in accumulation of abnormal mitochondria, leading to compromised self-renewal, pluripotency, and differentiation [34, 35]. Bcl-2 nineteen-kilodalton interacting protein (BNIP3) is an atypical member of the pro-apoptotic Bcl-2 subfamily that contains a Bcl-2 homology domain (BH3) and transmembrane domain (TM) that targets BNIP3 to mitochondria, a PEST domain that controls BNIP3 degradation, and a conserved domain [36–38]. In contrast to typical BH3-only proteins that bind to anti-apoptotic Bcl-2 family proteins via their BH3 domain, BNIP3 interacts with Bcl-2 and Bcl-X_L_ specifically through its TM domain and N-terminus in cancer and non-cancer somatic cells [39]. The BNIP3 TM domain mediates the formation of homodimers that induce pro-survival mitophagy or cell death resembling both necrosis and apoptosis [39–43], but the interplay between BNIP3-induced apoptosis and survival functions remains obscure. Recently, we found that BNIP3-dependent mitophagy maintains mitochondrial homeostasis and pluripotency in mouse ESC [44]. However, how does BNIP3-dependent mitophagy regulate pluripotency remain obscure.

Here we uncovered that BNIP3-mediated mitophagy maintains ESC identity by preventing ESCs from oxidative stress-induced DNA damage and the resultant p53-dependent differentiation, while also maintains ESC genomic stability via protecting DNA repair by homologous recombination.

## Materials and methods

### Animals

GFP-LC3 mice (RBRC00806) [45] and Mito-RFP mice (RBRC03743) [46] were purchased from the Riken BioResource Centre. Dr. Zhuohua Zhang provided Bnip3 heterozygous mice. All protocols used for animal manipulation were approved by the Institutional Animal Care Committee.

### Reagents

MitoTracker Red (40743ES50) was purchased from Yeasen. DCFH-DA (D6883) was purchased from Sigma Aldrich. AICAR (S1802), Compound C (S7306), and Oligomycin A (S1478) were purchased from Selleck. 3-MA (M9281) and Baf-A1(B1793) were obtained from Sigma Aldrich.

### Antibodies

The following antibodies were used: anti-Bnip3 polyclonal antibody (3769, Cell Signaling Technology), anti-Actin monoclonal antibody (A5441, Sigma Aldrich), anti-LC3B antibody (PM036, Medical and Biological Laboratories, Co.), anti-Sox2 monoclonal antibody (ab92494, Abcam, for western blotting), anti-Sox2 polyclonal antibody (AF2018, R&D, for IF), anti-Oct4 polyclonal antibody (ab19857, Abcam), anti-Nanog polyclonal antibody (ab106465, Abcam), anti-γH2AX polyclonal antibody (CST7918, Cell Signaling Technology), anti-53BP1 polyclonal antibody (NB100-304, Bio Techne), anti-p-ATM (Ser1981) monoclonal antibody (CST13050, Cell Signaling Technology), anti-ATM monoclonal antibody (CST2873, Cell Signaling Technology), anti-p-p53(Ser15) monoclonal antibody (CST9284, Cell Signaling Technology), anti-p53 monoclonal antibody (CST2527, Cell Signaling Technology), anti-Rad51 monoclonal antibody (H00005888, Abnova), anti-p-AMPK(Thr172) (CST2531, Cell Signaling Technology), anti-p-AMPK (CST2532, Cell Signaling Technology), anti-SSEA-1 monoclonal antibody (SC-21702AF647, Santa Cruz Biotechnology), Alexa Fluor 488 donkey anti-rabbit IgG (A21206, Invitrogen Thermo Fisher Scientific), Alexa Fluor 594 donkey anti-mouse IgG (A 21203, Invitrogen Thermo Fisher Scientific), Alexa Fluor 647 donkey anti-goat IgG (A32849, Invitrogen Thermo Fisher Scientific).

### Plasmids

pMXs-Oct4, pMXs-Sox2, pMXs-Klf4, and pMXs-cMyc were purchased from Addgene (13366, 13367, 13370, 13375; Deposited by the Shinya Yamanaka lab). Mito-Keima vectors were shared by Dr. Quan Chen. Bnip3 and its mutants were cloned into pMXs and pCDH-CAG-RFP lenti-vectors as described previously [47]. ShRNA were designed and cloned into pSicor-GFP or pSicor vectors. The NHEJ vector, HR vector, and I-SceI-expressing plasmid were shared by Dr. Lingyi Chen.

### ESC isolation and iPSC induction

Bnip3 knockout and wild-type ESCs were isolated at E3.5 and seeded on feeder layers for selection using 2i medium. Then ESCs were cultured for 3 to 5 passages and maintained in ESC medium (knockout DMEM with 15% fetal bovine serum, 2 mM glutamine, 1 mM sodium pyruvate, 0.1 mM nonessential amino acids, 100 mg/ml streptomycin, and 100 U/ml penicillin, 0.055 mM β-mercaptoethanol, and 1000 U/ml leukemia inhibitory factor). For iPSC induction, MEFs were seeded at 50,000 cells/well in a 6-well plate and infected with a retrovirus cocktail expressing Oct4, Sox2, Klf4, and Myc; iPSC colonies were picked 14 d after infection as described previously [47].

### Generation of Bnip3 & ATM knockout and Bnip3 & p53 knockout ESCs

ATM and Bnip3 double knockout and p53 and Bnip3 double knockout ESCs were generated using a CRISPR-Cas9 system. ATM and p53 gRNA were cloned into the px330 vector, and transfected into Bnip3 knockout ESCs with a Gene pulser Xcell II (Bio-Rad) according to the manufacturer’s protocol. Then, knockout ESCs were identified by digestion with *Pst*I for ATM and *Nco*I for p53 and sequencing. All knockout ESCs were further identified by western blot.

### Western blot

Cells were lysed in RIPA buffer (50 mM Tris-HCl, pH 7.4, 150 mM NaCl, 0.5% sodium deoxycholate, 1% Nonidet P-40, 5 mM EGTA, 2 mM EDTA, 10 mM NaF) with protease inhibitor cocktail (04693116001, Roche). Equivalent protein quantities (20 µg) were subjected to SDS-PAGE and transferred to nitrocellulose membranes (Millipore), which were probed with the indicated primary antibodies followed by the appropriate HRP-conjugated secondary antibodies (Santa Cruz). Immunoreactive bands were detected with a Luminata Forte Western HRP Substrate Kit (WBLUF0100, Millipore).

### Immunofluorescence microscopy

For LC3-GFP and Mito-RFP immunostaining, we used GFP-LC3 and Mito-RFP MEFs. For Bnip3 immunostaining, cells were cultured on gelatin-coated glass slides and fixed with 4% PFA for 20 min. They were then washed with DPBS, permeabilized with 0.2% Triton-X100 for 0.5 h and blocked with 2% BSA for 1 h before staining with the appropriate primary antibody overnight at 4 °C and incubation with secondary antibody for 2 h at 37 °C. Cell nuclei were stained with DAPI. For Mito-Keima immunostaining, ESCs were transfected with Mito-Keima and directly monitored by confocal microscopy.

### Measurement of HR

HR measurement was carried out as described [48]. We transfected the vector into *Bnip3*^*+/+*^ ESCs, *bnip3*^*−/−*^ ESCs and rescued ESCs, then selected stably expressing clones with puromycin. To induce DSB, we transfected 1 μg I-SceI–expressing plasmid into HR reporter ESC lines. ESCs were harvested for FACS analysis 48 h after infection.

### Mito-Keima assay

Mito-Keima was used to monitor mitophagy as described [49, 50]. Mito-Keima was transfected into *Bnip3*^*+/+*^ ESCs, *bnip3*^*−/−*^ ESCs, and rescued ESCs. Stably expressing clones were sorted by FACS. The bnip3-rescued vectors were transfected into the above cell lines and single clones were picked for western blot identification. ESCs were cultured under normal conditions or treated with FCCP for 3 h, and then we detected the above cell lines by confocal laser scanning microscopy for Mito-Keima imaging [51].

### Transmission electron microscopy

Cells were fixed in 2.5% glutaraldehyde for 2 h at 4 °C and immersed in 2% osmium tetroxide. The samples were then sequentially dehydrated with 50, 70, 90, 95, and 100% ethanol. Copper grids were used for ultrathin section collection and uranyl acetate and lead citrate were used for counterstaining. Images were taken using a FEI Tecnai Spirit transmission electron microscope.

### Measurement of ATP and ROS

A CellTiter-GloLuminescent Cell Viability Assay kit (Promega Corporation, 0000092970) was used to assess cellular ATP content. Cellular ROS was measured by flow cytometry using HDCF-DA from Sigma Aldrich (D6883).

### Measurement of OCR and ECAR

Seahorse XF24 analyzer (Seahorse Bioscience Asia, Shanghai) was used for detecting OCR and ECAR in ESCs or iPSCs. Briefly, ESCs or iPSCs were seeded at 60,000 cells/well for 6 h, and measurements were made in strictly accordance with the standard protocol in the manual. ATP contribution was calculated under the manual.

### AP staining and clone formation assay

For AP staining, a BCIP/NBT Alkaline Phosphatase Colour Development Kit (Beyotime) was used according to the manufacturer’s instructions. The clone formation assay was used as described. Briefly, ESCs were trypsinized into single cells that were seeded into 6-well plates (coated with feeder) at 1000 cells/well and cultured for one week. The number of AP-positive cells was counted after AP staining.

## Results

### Inhibition of autophagy increases DNA damage response in mouse ESCs

**Fig. 1:**
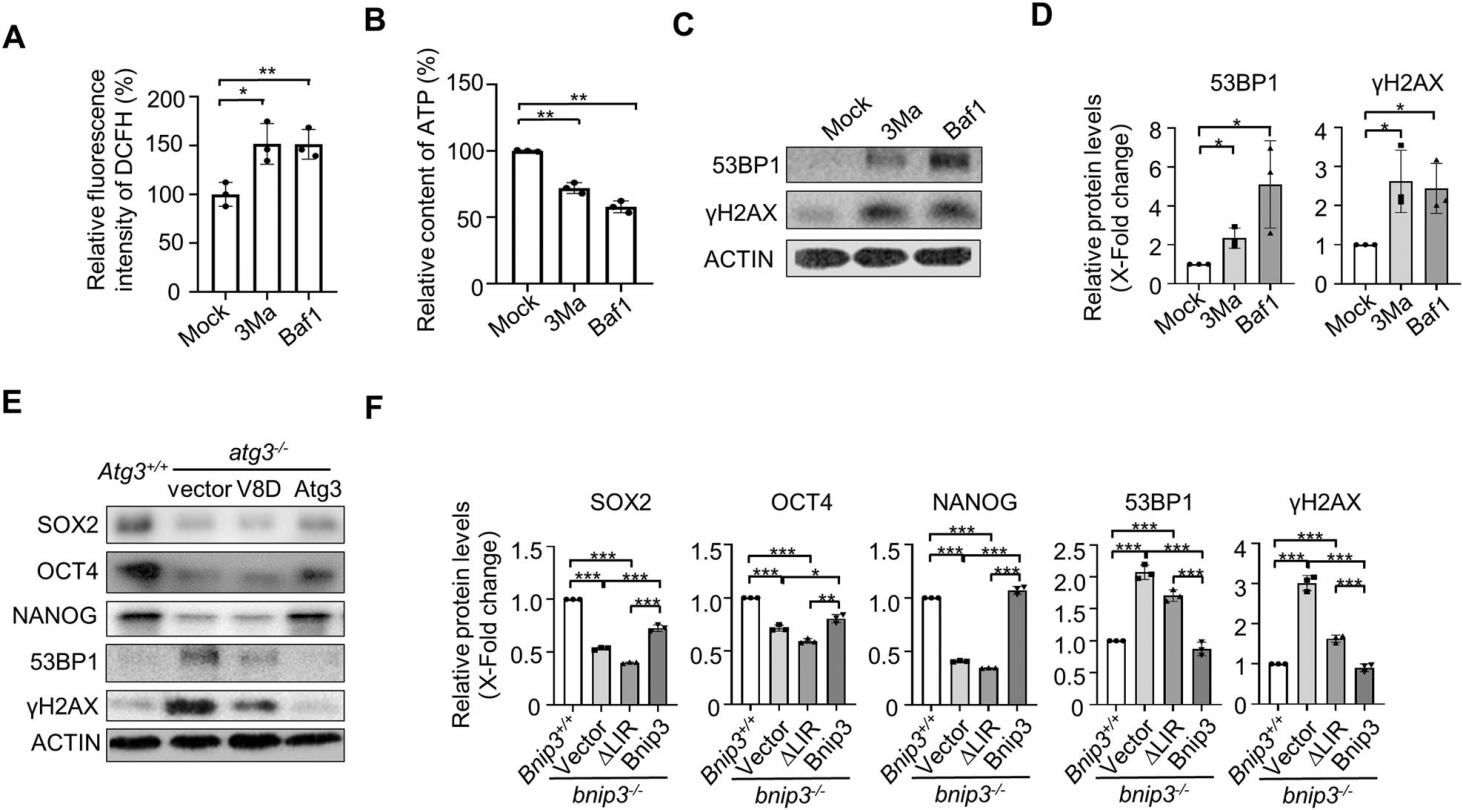
Inhibition of autophagy increases DNA damage in mouse ESCs. **A** Treatment of ESCs with 3-MA or Baf1 increased ROS generation. Data shown are the mean ± SD, *n* = 3; *, *P* < 0.05; **, *P* < 0.01; Student’s t-test. **B** Treatment of ESCs with 3-MA or Baf1 decreased ATP generation. Data shown are the mean ± SD, *n* = 3; **, *P* < 0.01; Student’s t-test. **C** 3-MA or Baf1 treatment induced enhanced DNA damage response in ESCs. **D** Quantifications of each protein expression in (**C**). Data are shown as mean ± SD, *n* = 3; *, *P* < 0.05; Student’s t-test. **E** The decreased expression of SOX2, OCT4, NANOG and increased expression of 53BP1, γH2AX in *atg3*^*−/−*^ ESCs were rescued by gain expression of wide-type but not LC3-binding dead mutant V8D *Atg3*. **F** Quantifications of each protein expression in (**E**). Data are shown as mean ± SD, *n* = 3; *, *P* < 0.05; **, *P* < 0.01; ***, *P* < 0.001; Student’s t-test.

Previous study reported that loss of autophagy caused a synthetic lethal deficiency in DNA repair in mouse embryo fibroblasts (MEFs) via a checkpoint kinase 1 (Chk1) dependent manner [52]. ESCs failed to activate the ATR-Chk1 signaling axis under alkylation-induced DNA damage [53]. To test whether autophagy contributes to genomic stability regulation in ESCs, we inhibited autophagy by 3MA or Baf1 treatment in ESCs. Treatment of ESCs with either 3MA or Baf1 resulted in increased ROS production and decreased ATP generation (Fig. 1A, B). Correspondingly, DNA damage response in ESCs was dramatically enhanced as indicated by the elevation levels of 53BP1 and γH2AX upon 3MA or Baf1 treatment (Fig. 1C, D). Importantly, the enhanced DNA damage response in *atg3*^*−/−*^ ESCs could be rescued by gain expression of wide-type but not LC-3-binding dead mutant V8D *Atg3* (Fig. 1E, F), supporting ATG3 mediated autophagy contributes to DNA damage response in ESCs.

### BNIP3-dependent mitophagy safeguards ESC identity by preventing dysfunctional mitochondrion accumulation-induced oxidative stress and corresponding p53-mediated differentiation

**Fig. 2:**
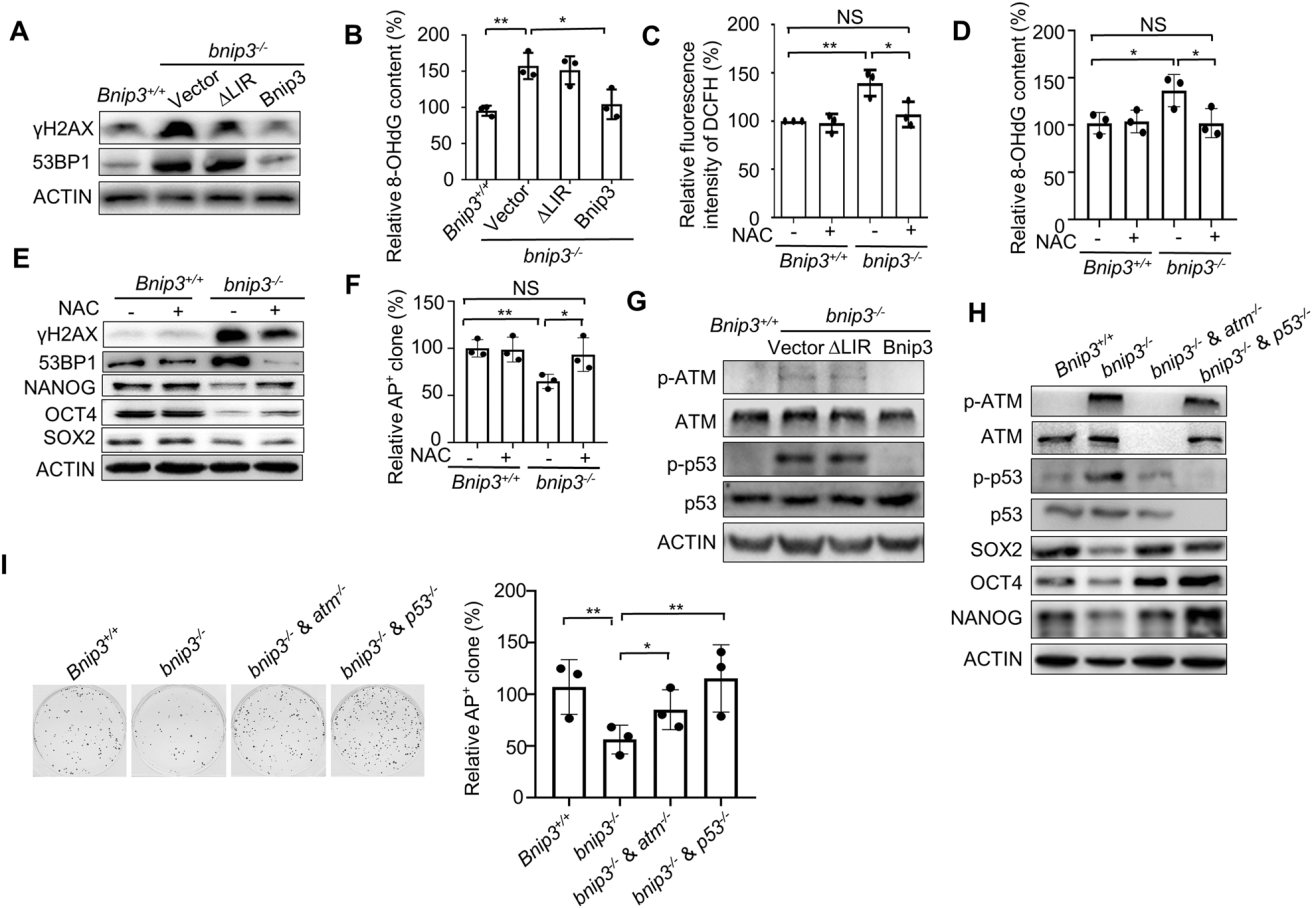
BNIP3-dependent mitophagy protects ESC from oxidative stress-induced DNA damage. **A** Western blot of γH2AX and 53BP1 in *bnip3*^*−/−*^ ESCs and rescued ESCs with actin as the loading control. **B** Increased cellular 8-OHdG in *bnip3*^*−/−*^ ESCs was rescued by gain of expression of wild-type but not ∆LIR *Bnip3*. Data shown are the mean ± SD, *n* = 3; *, *P* < 0.05; **, *P* < 0.01; Student’s t-test. **C** NAC treatment (100 μM for 12 h) suppressed elevated oxidative stress in *bnip3*^*−/−*^ ESCs. Data shown are the mean ± SD, *n* = 3; *, *P* < 0.05; **, *P* < 0.01; NS no significant difference, Student’s t-test. **D** NAC treatment antagonized increased DNA damage in *bnip3*^*−/−*^ ESCs. Data shown are the mean ± SD, *n* = 3; ^*,^ *P* < 0.05; NS no significant difference, Student’s t-test. **E** Compromised pluripotency gene expression and enhanced DNA damage in *bnip3*^*−/−*^ ESCs can be rescued by NAC treatment. Actin served as the loading control. **F** Deteriorated self-renewal of *bnip3*^*−/−*^ ESCs can be restored by NAC treatment. Data shown are the mean ± SD, *n* = 3; *, *P* < 0.05; **, *P* < 0.01; NS no significant difference, Student’s t-test. **G** Activation of ATM and p53 in *bnip3*^*−/−*^ ESCs can be rescued by wild-type but not ∆LIR *Bnip3*. Actin served as the loading control. **H** Decreased expression of pluripotency genes in *bnip3*^*−/−*^ ESCs can be rescued by knockout of either ATM or p53. Actin served as the loading control. **I** Compromised self-renewal in *bnip3*^*−/−*^ ESCs can be rescued by knockout of either ATM or p53. Data shown are the mean ± SD, *n* = 3; *P* < 0.01; Student’s t-test.

Recently we have uncovered BNIP3-mediated mitophagy maintains mouse ESC pluripotency by regulating mitochondrial homeostasis [44]. Knockout of *Bnip3* leads to enhanced ROS generation, decreased ATP production and impaired mitochondrial cristae without affecting cellular viability and apoptosis (Supplementary Fig. 1). Since *Bnip3* knockout is associated with significant increases in ROS generation, and excessive intracellular ROS levels can cause DNA damage, we next asked whether *Bnip3* depletion resulted in increased amounts of ROS-mediated DNA damage. In *bnip3*^*−/−*^ ESCs, γH2AX and 53BP1 expression, as well as levels of the oxidative stress-induced DNA damage indicator 8-OHdG, were significantly increased compared to wild-type ESCs (Fig. 2A, B and Supplementary Fig. 2A). These defects could be partially restored by gain of expression of wild-type but not ∆LIR *Bnip3* or addition of the antioxidant NAC (Fig. 2A–E and Supplementary Fig. 2B). Together, these data indicated that increased ROS generation induced by dysfunction of BNIP3-dependent mitophagy in ESCs directly caused genomic DNA damage.

We then assessed whether deterioration of self-renewal and pluripotency in *bnip3*^*−/−*^ ESCs was caused by ROS accumulation that occurred with *Bnip3* depletion. As expected, the decreased expression of pluripotency genes and compromised colony formation of *bnip3*^*−/−*^ ESCs were partially rescued by NAC treatment (Fig. 2E, F), supporting that the deterioration of *bnip3*^*−/−*^ ESCs results from dysfunctional BNIP3-dependent mitophagy induced oxidative stress.

To dissect the interplay between enhanced genomic DNA damage by *Bnip3* depletion and ESC identity, we examined downstream events of ROS accumulation, particularly the DNA damage response (DDR) in *bnip3*^*−/−*^ ESCs. We found that increased phosphorylation of ATM (Ser1981) and p53 (Ser15) in *bnip3*^*−/−*^ ESCs, which can be rescued by gain expression of wild-type but not ∆LIR *Bnip3*, could be restored by NAC treatment, indicating that intracellular ROS accumulation following *Bnip3* knockout contributed to activation of ATM and p53 (Fig. 2G and Supplementary Fig. 2C, D). Correspondingly, both colony formation ability and expression of pluripotency marker gens in *bnip3*^*−/−*^ ESCs showed significant defects, and colonies of *bnip3*^*−/−*^ ESCs exhibited differentiation phenotype (Fig. 2H, I and Supplementary Fig. 4A–C). Then, we knocked out ATM or p53 in *bnip3*^*−/−*^ ESCs (Supplementary Fig. S3). As expected, decreased expression of pluripotency genes, compromised self-renewal capability, as well as the differentiation phenotype of *bnip3*^*−/−*^ ESCs were compensated by knockout of either ATM or p53 (Fig. 2H, I and Supplementary Fig. 4A–C). Together, these data suggested that BNIP3-mediated mitophagy maintains ESC identity by preventing dysfunctional mitochondrion accumulation caused oxidative stress and subsequent p53-dependent differentiation.

### BNIP3-dependent mitophagy protects genomic integrity by safeguarding homologous recombination

**Fig. 3:**
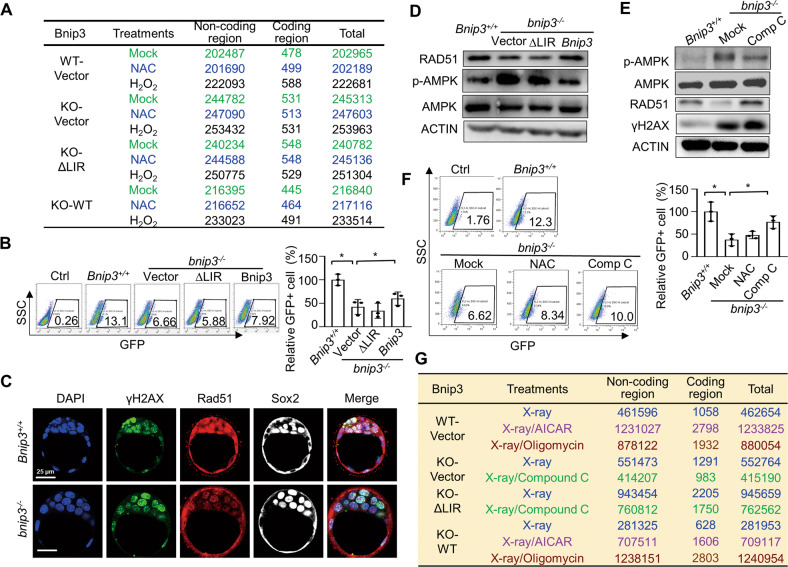
BNIP3-dependent mitophagy safeguards homologous recombination. **A** Acquired indels (insertions-deletions) in Vector-*Bnip3*^*+/+*^, Vector-*binp3*^*−/−*^, ∆LIR-*binp3*^*−/−*^ and WT-*binp3*^*−/−*^ ESC lines after 30 passages with the indicated treatments. Whole genome sequencing of Vector-*Bnip3*^*+/+*^, Vector-*binp3*^*−/−*^, ∆LIR-*binp3*^*−/−*^*,* and WT-*binp3*^*−/−*^ ESC lines were performed and acquired indels were showed in table. Data shown are one representative of biological replicates. **B** Deteriorated HR in *binp3*^*−/−*^ ESCs was partially rescued by wild-type but not mutant ∆LIR *Bnip3*. Data shown are the mean ± SD, *n* = 3; *, *P* < 0.05; Student’s *t*-test. **C** Decreased colocalization of γH2AX and RAD51 in the inner cell mass of *bnip3*^*−/−*^ blastocysts. Green, γH2AX; Red, RAD51; White, SOX2; Blue, DAPI. **D** Excessive AMPK activation and decreased RAD51 expression in *binp3*^*−/−*^ ESCs was partially rescued by wild-type but not ∆LIR *Bnip3*. **E** Inactivation of AMPK by Compound C restored compromised expression of RAD51 in *binp3*^*−/−*^ ESCs without affecting γH2AX expression. Actin served as the loading control. **F** Compromised HR in *binp3*^*−/−*^ ESCs was partially rescued by adding AMPK inhibitor Compound C, but not antioxidant NAC. Data shown are the mean ± SD, *n* = 3; *, *P* < 0.05; Student’s *t*-test. **G** Acquired indels in Vector-*Bnip3*^*+/+*^, Vector-*binp3*^*−/−*^, ∆LIR-*binp3*^*−/−*^ and WT-*binp3*^*−/−*^ ESCs with indicated treatments. Whole genome sequencing of Vector-*Bnip3*^*+/+*^, Vector-*binp3*^*−/−*^, ∆LIR-*binp3*^*−/−*^, and WT-*binp3*^*−/−*^ ESC lines were performed and acquired indels were shown in table. Data shown are one representative of biological replicates.

In addition to compromised self-renewal and pluripotency associated with DDR*, Bnip3*-knockout ESCs form colonies with lower efficiency, providing an ideal model to evaluate long-term effects of excessive ROS on ESC genomic stability. We next investigated whether increased levels of intracellular ROS in *bnip3*^*−/−*^ ESCs would significantly impair genomic integrity during long-term propagation. We propagated *Bnip3*^*+/+*^, vector-*bnip3*^*−/−*^, ∆LIR-*bnip3*^*−/−*^, and WT-*bnip3*^*−/−*^ ESCs routinely for 30 passages either under normal conditions or with NAC or H_2_O_2_ treatment. Then we performed whole genomic sequencing on each cell line at either initial passage or after propagation for 30 passages. Compared to *Bnip3*^*+/+*^ ESCs, the *bnip3*^*−/−*^ ESC genome accumulated more mutations during long-term propagation (Fig. 3A). Mitochondrial genome of *Bnip3*^*+/+*^ ESCs showed similar mutation rates with that of *bnip3*^*−/−*^ ESCs. This enhanced accumulation of mutations in *bnip3*^*−/−*^ ESCs was partially rescued by wild-type but not ∆LIR-*Bnip3*, indicating that BNIP3-dependent mitophagy protects genomic stability during long-term propagation of ESCs (Fig. 3A).

As expected, H_2_O_2_ treatment during long-term propagation further increased genomic mutation load in *Bnip3*^*+/+*^, vector-*bnip3*^*−/−*^, ∆LIR-*bnip3*^*-/-*^, and WT-*bnip3*^*−/−*^ ESCs. Unexpectedly, however, NAC treatment of vector-*bnip3*^*−/−*^ or ∆LIR-*bnip3*^*−/−*^ ESCs during long-term propagation did not mitigate the elevated genomic mutation load (Fig. 3A). These data suggest that increased ROS levels are likely not the direct cause of enhanced genomic mutation load observed in *bnip3*^*−/−*^ ESCs during long-term propagation. To dissect this observation further, we assessed DNA repair by homologous recombination (HR) in *Bnip3*^*+/+*^, vector-*bnip3*^*−/−*^, ∆LIR-*bnip3*^*−/−*^, and WT-*bnip3*^*−/−*^ ESCs. Surprisingly, we found that depletion of *Bnip3* significantly decreased the HR rate (Fig. 3B). These defects were also observed in developing *bnip3*^*−/−*^ blastocysts (Fig. 3C). The compromised HR in *bnip3*^*−/−*^ ESCs was partially compensated by gain of expression of wild-type, but not the LIR-binding deficient ∆LIR *Bnip3* (Fig. 3B). These data indicate that BNIP3-dependent mitophagy protects mouse ESC genomic stability by safeguarding HR.

### Excessive AMPK activation decreases homologous recombination

Adenosine monophosphate (AMP)-activated protein kinase (AMPK) is a cellular energy sensor that is regulated by both cellular adenine nucleotides and glucose [54–56]. As shown above, *Bnip3* depletion resulted in dysfunctional mitochondrial accumulation and significantly decreased cellular ATP generation by oxidative phosphorylation (Supplementary Fig. 5A) [44]. Although mouse ESCs had higher expression levels of AMPK and enhanced AMPK phosphorylation than somatic fibroblasts constitutively [57], the lower rate of ATP generation in *bnip3*^*−/−*^ ESCs further enhanced AMPK phosphorylation without affecting AMPK expression, which was rescued by gain of expression of wild-type but not ∆LIR-*Bnip3* (Fig. 3D and Supplementary Fig. 5B). We next asked whether excessive AMPK activation in *bnip3*^*−/−*^ ESCs is responsible for the defective HR and associated increase in DNA mutation accumulation. Inhibition of excessive AMPK activation with Compound C in *bnip3*^*−/−*^ ESCs partially restored the compromised HR activity without affecting DNA damage (Fig. 3E, F and Supplementary Fig. 5C). In contrast, treatment of *Bnip3*^*+/+*^ ESCs with the AMPK agonist AICAR or the mitochondrial respiration inhibitor oligomycin compromised DNA repair by HR (Supplementary Fig. 5D). These data support that excessive activation of AMPK did result in impaired HR DNA repair in mouse ESCs.

We next tested whether excessive activation of AMPK could diminish ESC genomic integrity. Both *Bnip3*^*+/+*^ and WT-*bnip3*^*−/−*^ ESCs were treated with X-ray irradiation in the presence or absence of AICAR or oligomycin. Both the AMPK agonist and mitochondrial inhibitor significantly increased the genomic mutation load in *Bnip3*^*+/+*^ ESCs, indicating that excessive AMPK activation caused by mitochondrial dysfunction in ESCs deteriorated genomic stability (Fig. 3G). In addition, we treated vector-*bnip3*^*−/−*^ and ∆LIR-*bnip3*^*−/−*^ ESCs with X-rays and the AMPK inhibitor Compound C, and found that inhibition of excessive AMPK activation in *bnip3*^*−/−*^ ESCs dramatically decreased the genomic mutation load (Fig. 3G). At the same time, we evaluated the genome stability of *Bnip3*^*+/+*^ and *bnip3*^*−/−*^ ESCs after 30 passages in the presence or absence of Compound C by detecting the DNA damage maker and HR maker via western blot. We found the surviving *bnip3*^*−/−*^ ESCs showed enhanced DNA damage with no DNA repair changes after 30 passages. Long-term culturing led to increased DNA damage in both *Bnip3*^*+/+*^ and *bnip3*^*−/−*^ ESCs. Compound C treatments significantly enhanced DNA repairs in both *Bnip3*^*+/+*^ and *bnip3*^*−/−*^ ESCs (Supplementary Fig. 5E, F). Together, these data suggested that enhanced accumulation of genomic mutations is due to excessive AMPK activation induced by deterioration of mitochondrial bioenergetics in *bnip3*^*−/−*^ ESCs.

### Enhancement of iPSC genomic integrity by increasing BNIP3-mediated mitophagy

**Fig. 4:**
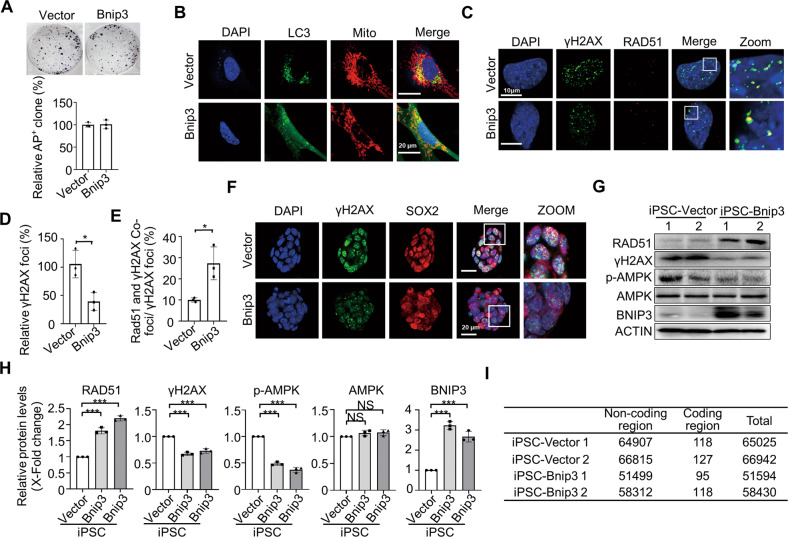
Bnip3 overexpression improves iPSC genomic integrity. **A** Overexpression of *Bnip3* does not affect reprogramming efficiency. Data shown are the mean ± SD, *n* = 3; NS no significant difference. **B** *Bnip3* overexpression enhanced mitochondrial autophagy in reprogramming cells. GFP: LC3; RFP: Mitochondria. **C** Confocal fluorescence micrographs of reprogramming cells. SSEA-1-positive cells at reprogramming day 3 were sorted for staining with γH2AX and RAD51. Green, γH2AX; Red, RAD51; Blue, DAPI. **D** Statistical data for γH2AX foci in (**C**). Cells were randomly divided into 3 groups and the foci were counted. Data shown are the mean ± SD, *n* = 3; *, *P* < 0.05; Student’s t-test. **E** Statistical data for RAD51 and γH2AX colocalization foci/γH2AX foci in (**C**). **F** Confocal fluorescence micrographs of iPSCs. Green, γH2AX; Red, SOX2; Blue, DAPI. **G** Western blot detection of RAD51, γH2AX, p-AMPK, AMPK, and BNIP3 in wild-type or Bnip3-overexpressing iPSCs. Actin served as the loading control. **H** Quantifications of each protein expression in (**G**). Data are shown as mean ± SD, *n* = 3; ***, *P* < 0.001; NS no significant difference, Student’s t-test. **I** Acquired indels in indicated iPSC lines.

Given that BNIP3 has a role in protecting genomic integrity of ESCs, we tested whether enhancement of BNIP3-dependent mitophagy during reprogramming improves genomic stability of iPSCs. *Bnip3* overexpression significantly enhanced mitochondrial autophagy during reprogramming without affecting reprogramming efficiency (Fig. 4A, B). By sorting of SSEA-1 positive cells on reprogramming day 3, we found that *Bnip3* overexpression significantly alleviated ROS production (Supplementary Fig. 6A, B). Correspondingly, DDR activation, as evidenced by the presence of γH2AX foci, was significantly decreased in cells overexpressing *Bnip3* compared to WT reprogramming cells (Fig. 4C). Importantly, activation of DNA repair was dramatically increased in cells overexpressing *Bnip3* compared with WT reprogramming cells (Fig. 4D, E). These data suggested that enhancement of BNIP3-dependent mitophagy by overexpression of *Bnip3* during reprogramming lessened oxidative stress-induced DDR and increased DNA repair by HR. Thus, we would expect to see improved genetic integrity in the resulting iPSCs having *Bnip3* overexpression. Correspondingly, the established iPSCs with *Bnip3* overexpression showed decreased ROS levels and enhanced mitochondrial respiration (Supplementary Fig. 6C–G). Supportively, the iPSCs with *Bnip3* overexpression exhibited decreased DNA damage and AMPK activation, while enhanced DNA repair (Fig. 4F–H). Whole genomic sequencing on established iPSC lines carrying either empty vector or *Bnip3* cDNA, and their parental fibroblasts, demonstrated that *Bnip3* overexpression indeed dramatically reduced the mutation load in established iPSCs (Fig. 4I).

## Discussion

**Fig. 5:**
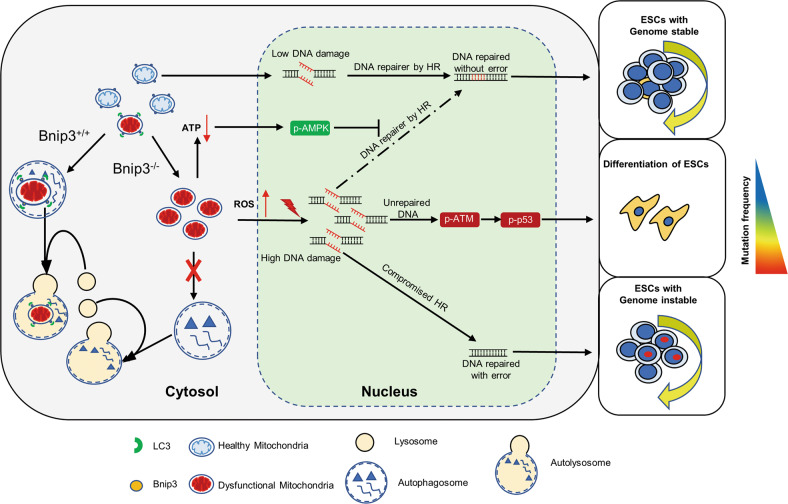
BNIP3-dependent mitophagy safeguards ESC genomic integrity. Dysfunction of BNIP3-dependent mitophagy induces cellular ROS accumulation that leads to p53-mediated ESC differentiation, and decreased ATP generation that results in compromised AMPK-dependent homologous recombination. Reinforcement of BNIP3-dependent mitophagy during reprogramming improves iPSC genomic integrity.

In this study, we revealed that BNIP3-mediated mitophagy maintains ESC identity by ensuring mitochondrial integrity that avoids excessive ROS generation and the corresponding p53-dependent ESC differentiation. Meanwhile, BNIP3 protects ESC genomic stability by safeguarding homologous recombination through maintenance of mitochondrial bioenergetics. In addition, reinforcement of BNIP3-dependent mitophagy during reprogramming is demonstrated to be an alternate strategy to improve iPSC genomic stability (Fig. 5).

Excessive levels of cellular ROS, the byproducts of mitochondrial oxidative phosphorylation, are detrimental to genomic DNA, proteins, and lipids [58]. Previous studies showed that ESC mitochondria are in an immature state characterized by a globular shape, blurred cristae, and limited respiration capability compared to differentiated somatic cells [59–64]. Somatic cells utilize oxidative phosphorylation, whereas ESCs are almost entirely glycolytic with low mitochondrial activity under aerobic conditions [22, 64]. In agreement with these mitochondrial characteristics, ESCs produce less ROS and have a higher antioxidant capacity compared to somatic cells [19, 65, 66]. Thus, it was proposed that lower cellular ROS serves as a defense mechanism for ESCs to maintain genomic integrity. In contrast, here we showed that increased cellular ROS levels that accompany *Bnip3* depletion resulted in p53 activation that promoted ESC differentiation and corresponding compromises in self-renewal. Treatment with NAC or knockout of p53 in *Bnip3*^*−/−*^ ESCs compensate for the compromised self-renewal, highlighting the relationships between *Bnip3*-knockout-induced ROS accumulation, p53 activation, and maintenance of ESC identity. Interestingly, a recent study showed that loss of *Cops*5, conserved COP9 signalosome subunit 5, resulted in increased ROS generation, p53 activation, G2/M arrest, and apoptosis rather than p53-dependent differentiation in mouse ESC [48]. The mechanisms for these differential effects of ROS on determination of ESC fate are unclear. Existing studies have proposed that signal transduction of ROS-induced p53 activation may depend on the extent of DNA damage, since higher levels of DNA double-stranded break (DSB) damage induce both p53-dependent and p53-independent apoptosis, whereas low levels of DNA damage suppress p53-dependent expression of critical pluripotency genes like Nanog and Oct4 [29].

Treatment of *Bnip3*^*+/+*^ ESCs with H_2_O_2_ further increased genomic mutation load during long-term propagation; however, NAC treatment did not decrease genomic mutation accumulation in the same timeframe (Fig. 3A). Intriguingly, treatment of *Bnip3*^*−/−*^ ESCs with NAC had little impact on genomic mutation load during long-term propagation of these cells (Fig. 3A), although it could restore the defective self-renewal ability (Fig. 2F). These data suggest that BNIP3-dependent mitophagy does not directly rely on ROS homeostasis maintenance function to protect ESC genomic integrity. Instead, ESCs developed a fail-safe elimination mechanism to maintain genomic stability by inducing p53-dependent differentiation of cells having elevated ROS levels to remove them from self-renewing pools.

Genomic DNA can be damaged by exogenous stresses like UV light, ionizing radiation, and chemical compounds, as well as by endogenous stress like ROS, and DNA replication and transcription activities [67, 68]. Mammalian cells have developed sophisticated mechanisms to protect their genomic integrity. After DNA damage, cells quickly initiate DDR to induce cell-cycle arrest, apoptosis, or senescence [15]. In contrast to somatic cells, PSCs have a short G1 phase and no G1/S checkpoint, which possibly causes constitutive replication stresses like single-stranded DNA gap accumulation and corresponding constitutive activation of DDR as characterized by elevated phospho-H2AX [24, 69]. Earlier studies showed that PSCs preferentially employ HR over NHEJ, thus repairing DNA DSBs with enhanced fidelity [23]. At the same time, expression of some DNA repair machinery components has been found to be significantly increased in PSCs compared to somatic cells [17, 65]. In addition, ESC-specific factors like ZSCAN4, FILIA, COPS5, and SALL4 have been shown to regulate pluripotency and genomic integrity coordinately [48, 70–72]. In contrast, here we demonstrated a novel pathway in mouse ESCs that connects mitochondrial integrity and genomic stability through BNIP3-dependent mitophagy that maintains genomic stability by safeguarding DNA repair. Dysfunction of BNIP3-dependent mitophagy caused the accumulation of abnormal mitochondria and in turn, decreased ATP generation. The resulting decreased intracellular ATP levels of *Bnip3*^*−/−*^ ESCs further activate AMPK, leading to compromised DNA repair by HR. Interestingly, we previously showed that AMPK is highly expressed in ESCs relative to somatic fibroblasts, and that phosphorylation of ULK1 by AMPK is essential for mESC self-renewal and maintenance of pluripotency [57]. These data together suggest that appropriate activation of AMPK is required for ESCs to maintain cellular homeostasis and identity, whereas over-activation of AMPK in ESCs is detrimental to genomic stability. Indeed, chemical activation of AMPK in X-ray-stressed wild-type ESCs significantly enhanced the genomic mutation load, while AMPK inactivation in X-ray-stressed *Bnip3*^*−/−*^ ESCs ameliorated genome DNA damage (Fig. 3G).

Reprogramming of somatic cells to iPSCs is an inefficient process. Suppressed expression of the tumor suppressor gene p53 significantly enhances reprogramming efficiency, due to decreased p53-dependent DNA damage responses and induction of apoptosis [15, 73–77]. The potential need for p53 inactivation for successful reprogramming raises concerns about genomic stability of iPSCs and genomic structure variations have, in fact, been identified in multiple iPSC lines [9, 78, 79]. More than half of the somatic coding mutations present in 22 human iPS cell lines analyzed were found to be acquired during the reprogramming process, independently of the reprogramming method used [80]. These findings concerning genomic instability of iPSCs pose safety concerns for the clinical development iPSC technology[81]. In this regard, our results suggest an alternate strategy wherein reinforcement of BNIP3-dependent mitophagy during reprogramming could be used to improve iPSC genomic stability and facilitate clinical application of these cells.

### Data deposition

Whole genome sequencing data of Vector-Bnip3^+/+^, Vector-binp3^*−/−*^, ∆LIR-binp3^*−/−*^ and WT-binp3^*−/−*^ ESC were uploaded to Science Data Bank. The CSTR of our genome sequencing data is 31253.11.sciencedb.02743, and the DOI is 10.57760/sciencedb.02743. The details of data are available from the corresponding author on reasonable request.

## Supplementary information

Supplemental Figure legends

Supplementary Figure 1

Supplementary Figure 2

Supplementary Figure 3

Supplementary Figure 4

Supplementary Figure 5

Supplementary Figure 6

Original Data File

checklist

## Data Availability

The data and materials during the current study are available from the corresponding author on reasonable request.

## References

[1] Liu K, Song Y, Yu H, Zhao T (2014). Understanding the roadmaps to induced pluripotency. Cell Death Dis.

[2] Yu J, Vodyanik MA, Smuga-Otto K, Antosiewicz-Bourget J, Frane JL, Tian S (2007). Induced pluripotent stem cell lines derived from human somatic cells. Science..

[3] Takahashi K, Tanabe K, Ohnuki M, Narita M, Ichisaka T, Tomoda K (2007). Induction of pluripotent stem cells from adult human fibroblasts by defined factors. Cell.

[4] Takahashi K, Yamanaka S (2006). Induction of pluripotent stem cells from mouse embryonic and adult fibroblast cultures by defined factors. Cell..

[5] Thomson JA, Itskovitz-Eldor J, Shapiro SS, Waknitz MA, Swiergiel JJ, Marshall VS (1998). Embryonic stem cell lines derived from human blastocysts. Science..

[6] Evans MJ, Kaufman MH (1981). Establishment in culture of pluripotential cells from mouse embryos. Nature..

[7] Merkle FT, Ghosh S, Kamitaki N, Mitchell J, Avior Y, Mello C (2017). Human pluripotent stem cells recurrently acquire and expand dominant negative P53 mutations. Nature..

[8] Kilpinen H, Goncalves A, Leha A, Afzal V, Alasoo K, Ashford S (2017). Common genetic variation drives molecular heterogeneity in human iPSCs. Nature..

[9] Martins-Taylor K, Nisler BS, Taapken SM, Compton T, Crandall L, Montgomery KD (2011). Recurrent copy number variations in human induced pluripotent stem cells. Nat Biotechnol.

[10] International Stem Cell I, Amps K, Andrews PW, Anyfantis G, Armstrong L, Avery S (2011). Screening ethnically diverse human embryonic stem cells identifies a chromosome 20 minimal amplicon conferring growth advantage. Nat Biotechnol.

[11] Newman AM, Cooper JB (2010). Lab-specific gene expression signatures in pluripotent stem cells. Cell Stem Cell.

[12] Thompson O, von Meyenn F, Hewitt Z, Alexander J, Wood A, Weightman R (2020). Low rates of mutation in clinical grade human pluripotent stem cells under different culture conditions. Nat Commun.

[13] Cooper DJ, Chen IC, Hernandez C, Wang Y, Walter CA, McCarrey JR (2017). Pluripotent cells display enhanced resistance to mutagenesis. Stem Cell Res.

[14] Desmarais JA, Hoffmann MJ, Bingham G, Gagou ME, Meuth M, Andrews PW (2012). Human embryonic stem cells fail to activate CHK1 and commit to apoptosis in response to DNA replication stress. Stem Cells.

[15] Zhao T, Xu Y (2010). p53 and stem cells: New developments and new concerns. Trends Cell Biol.

[16] Stambrook PJ, Tichy ED (2010). Preservation of genomic integrity in mouse embryonic stem cells. Adv Exp Med Biol.

[17] Maynard S, Swistowska AM, Lee JW, Liu Y, Liu ST, Da Cruz AB (2008). Human embryonic stem cells have enhanced repair of multiple forms of DNA damage. Stem Cells.

[18] Hong Y, Cervantes RB, Tichy E, Tischfield JA, Stambrook PJ (2007). Protecting genomic integrity in somatic cells and embryonic stem cells. Mutat Res.

[19] Saretzki G, Armstrong L, Leake A, Lako M, von Zglinicki T (2004). Stress defense in murine embryonic stem cells is superior to that of various differentiated murine cells. Stem Cells.

[20] Cervantes RB, Stringer JR, Shao C, Tischfield JA, Stambrook PJ (2002). Embryonic stem cells and somatic cells differ in mutation frequency and type. Proc Natl Acad Sci USA.

[21] Fu X, Cui K, Yi Q, Yu L, Xu Y (2017). DNA repair mechanisms in embryonic stem cells. Cell Mol Life Sci: CMLS.

[22] Xu X, Duan S, Yi F, Ocampo A, Liu GH, Izpisua Belmonte JC (2013). Mitochondrial regulation in pluripotent stem cells. Cell Metab.

[23] Tichy ED, Pillai R, Deng L, Liang L, Tischfield J, Schwemberger SJ (2010). Mouse embryonic stem cells, but not somatic cells, predominantly use homologous recombination to repair double-strand DNA breaks. Stem Cells Dev.

[24] Ahuja AK, Jodkowska K, Teloni F, Bizard AH, Zellweger R, Herrador R (2016). A short G1 phase imposes constitutive replication stress and fork remodelling in mouse embryonic stem cells. Nat Commun.

[25] Momcilovic O, Knobloch L, Fornsaglio J, Varum S, Easley C, Schatten G (2010). DNA damage responses in human induced pluripotent stem cells and embryonic stem cells. PLoS One.

[26] Chuykin IA, Lianguzova MS, Pospelova TV, Pospelov VA (2008). Activation of DNA damage response signaling in mouse embryonic stem cells. Cell Cycle.

[27] Liu JC, Guan X, Ryan JA, Rivera AG, Mock C, Agrawal V (2013). High mitochondrial priming sensitizes hESCs to DNA-damage-induced apoptosis. Cell Stem Cell.

[28] Qin H, Yu T, Qing T, Liu Y, Zhao Y, Cai J (2007). Regulation of apoptosis and differentiation by p53 in human embryonic stem cells. J Biol Chem.

[29] Lin T, Chao C, Saito S, Mazur SJ, Murphy ME, Appella E (2005). p53 induces differentiation of mouse embryonic stem cells by suppressing Nanog expression. Nat Cell Biol.

[30] Chao C, Saito S, Kang J, Anderson CW, Appella E, Xu Y (2000). p53 transcriptional activity is essential for p53-dependent apoptosis following DNA damage. EMBO J.

[31] Chao C, Saito S, Anderson CW, Appella E, Xu Y (2000). Phosphorylation of murine p53 at ser-18 regulates the p53 responses to DNA damage. Proc Natl Acad Sci USA.

[32] Aladjem MI, Spike BT, Rodewald LW, Hope TJ, Klemm M, Jaenisch R (1998). ES cells do not activate p53-dependent stress responses and undergo p53-independent apoptosis in response to DNA damage. Curr Biol.

[33] Klionsky DJ, Abdel-Aziz AK, Abdelfatah S, Abdellatif M, Abdoli A, Abel S (2021). Guidelines for the use and interpretation of assays for monitoring autophagy (4th edition)(1). Autophagy..

[34] Wang S, Xia P, Ye B, Huang G, Liu J, Fan Z (2013). Transient activation of autophagy via Sox2-mediated suppression of mTOR is an important early step in reprogramming to pluripotency. Cell Stem Cell.

[35] Liu K, Zhao Q, Liu P, Cao J, Gong J, Wang C (2016). ATG3-dependent autophagy mediates mitochondrial homeostasis in pluripotency acquirement and maintenance. Autophagy..

[36] Burton TR, Gibson SB (2009). The role of Bcl-2 family member BNIP3 in cell death and disease: NIPping at the heels of cell death. Cell Death Differ.

[37] Yasuda M, Theodorakis P, Subramanian T, Chinnadurai G (1998). Adenovirus E1B-19K/BCL-2 interacting protein BNIP3 contains a BH3 domain and a mitochondrial targeting sequence. The. J Biol Chem.

[38] Boyd JM, Malstrom S, Subramanian T, Venkatesh LK, Schaeper U, Elangovan B (1994). Adenovirus E1B 19 kDa and Bcl-2 proteins interact with a common set of cellular proteins. Cell..

[39] Ray R, Chen G, Vande Velde C, Cizeau J, Park JH, Reed JC (2000). BNIP3 heterodimerizes with Bcl-2/Bcl-X(L) and induces cell death independent of a Bcl-2 homology 3 (BH3) domain at both mitochondrial and nonmitochondrial sites. J Biol Chem.

[40] Bocharov EV, Pustovalova YE, Pavlov KV, Volynsky PE, Goncharuk MV, Ermolyuk YS (2007). Unique dimeric structure of BNip3 transmembrane domain suggests membrane permeabilization as a cell death trigger. J Biol Chem..

[41] Vande Velde C, Cizeau J, Dubik D, Alimonti J, Brown T, Israels S (2000). BNIP3 and genetic control of necrosis-like cell death through the mitochondrial permeability transition pore. Mol Cell Biol.

[42] Zhu Y, Massen S, Terenzio M, Lang V, Chen-Lindner S, Eils R (2013). Modulation of serines 17 and 24 in the LC3-interacting region of Bnip3 determines pro-survival mitophagy versus apoptosis. J Biol Chem.

[43] Hanna RA, Quinsay MN, Orogo AM, Giang K, Rikka S, Gustafsson AB (2012). Microtubule-associated protein 1 light chain 3 (LC3) interacts with Bnip3 protein to selectively remove endoplasmic reticulum and mitochondria via autophagy. J Biol Chem.

[44] Liu K, Zhao Q, Sun H, Liu L, Wang C, Li Z (2022). BNIP3 (BCL2 interacting protein 3) regulates pluripotency by modulating mitochondrial homeostasis via mitophagy. Cell Death Dis.

[45] Mizushima N, Yamamoto A, Matsui M, Yoshimori T, Ohsumi Y (2004). In vivo analysis of autophagy in response to nutrient starvation using transgenic mice expressing a fluorescent autophagosome marker. Mol Biol Cell.

[46] Hasuwa H, Muro Y, Ikawa M, Kato N, Tsujimoto Y, Okabe M (2010). Transgenic mouse sperm that have green acrosome and red mitochondria allow visualization of sperm and their acrosome reaction in vivo. Exp Anim.

[47] Liu K, Zhao Q, Liu P, Cao J, Gong J, Wang C, et al. ATG3-dependent autophagy mediates mitochondrial homeostasis in pluripotency acquirement and maintenance. Autophagy. 2016;12:1–9. 10.1080/15548627.2016.1212786 PMC5103358 27575019

[48] Li P, Gao L, Cui T, Zhang W, Zhao Z, Chen L (2020). Cops5 safeguards genomic stability of embryonic stem cells through regulating cellular metabolism and DNA repair. Proc Natl Acad Sci USA.

[49] Wang C, Liu K, Cao J, Wang L, Zhao Q, Li Z (2021). PINK1-mediated mitophagy maintains pluripotency through optineurin. Cell Prolif.

[50] Shirakabe A, Fritzky L, Saito T, Zhai P, Miyamoto S, Gustafsson ÅB (2016). Evaluating mitochondrial autophagy in the mouse heart. J Mol Cell Cardiol.

[51] Liu K, Li X, Li Z, Cao J, Li X, Xu Y, et al. Evaluating mitophagy in embryonic stem cells by using fluorescence-based imaging. Front Cell Dev Biol. 2022;10:910464. 10.3389/fcell.2022.910464 PMC9520453 36187486

[52] Liu EY, Xu N, O’Prey J, Lao LY, Joshi S, Long JS (2015). Loss of autophagy causes a synthetic lethal deficiency in DNA repair. Proc Natl Acad Sci USA.

[53] Gupta D, Lin B, Cowan A, Heinen CD (2018). ATR-Chk1 activation mitigates replication stress caused by mismatch repair-dependent processing of DNA damage. Proc Natl Acad Sci USA.

[54] Lin SC, Hardie DG (2018). AMPK: Sensing glucose as well as cellular energy status. Cell Metab.

[55] Zhang CS, Hawley SA, Zong Y, Li M, Wang Z, Gray A (2017). Fructose-1,6-bisphosphate and aldolase mediate glucose sensing by AMPK. Nature..

[56] Hardie DG, Carling D (1997). The AMP-activated protein kinase-fuel gauge of the mammalian cell?. Eur J Biochem.

[57] Gong J, Gu H, Zhao L, Wang L, Liu P, Wang F (2018). Phosphorylation of ULK1 by AMPK is essential for mouse embryonic stem cell self-renewal and pluripotency. Cell Death Dis.

[58] Sies H, Berndt C, Jones DP (2017). Oxidative stress. Annu Rev Biochem.

[59] Facucho-Oliveira JM, St John JC (2009). The relationship between pluripotency and mitochondrial DNA proliferation during early embryo development and embryonic stem cell differentiation. Stem Cell Rev Rep.

[60] Prigione A, Fauler B, Lurz R, Lehrach H, Adjaye J (2010). The senescence-related mitochondrial/oxidative stress pathway is repressed in human induced pluripotent stem cells. Stem Cells.

[61] Suhr ST, Chang EA, Tjong J, Alcasid N, Perkins GA, Goissis MD (2010). Mitochondrial rejuvenation after induced pluripotency. PLoS One.

[62] Folmes CD, Dzeja PP, Nelson TJ, Terzic A (2012). Metabolic plasticity in stem cell homeostasis and differentiation. Cell Stem Cell.

[63] Zhang J, Nuebel E, Daley GQ, Koehler CM, Teitell MA (2012). Metabolic regulation in pluripotent stem cells during reprogramming and self-renewal. Cell Stem Cell.

[64] Zhang J, Zhao J, Dahan P, Lu V, Zhang C, Li H (2018). Metabolism in pluripotent stem cells and early mammalian development. Cell Metab.

[65] Cooper DJ, Walter CA, McCarrey JR (2014). Co-regulation of pluripotency and genetic integrity at the genomic level. Stem Cell Res..

[66] Cho YM, Kwon S, Pak YK, Seol HW, Choi YM, Park DJ (2006). Dynamic changes in mitochondrial biogenesis and antioxidant enzymes during the spontaneous differentiation of human embryonic stem cells. Biochem Biophys Res Commun.

[67] Vitelli V, Galbiati A, Iannelli F, Pessina F, Sharma S, d’Adda (2017). Recent advancements in DNA damage-transcription crosstalk and high-resolution mapping of DNA breaks. Annu Rev Genomics Hum Genet.

[68] Gaillard H, Aguilera A (2016). Transcription as a threat to genome integrity. Annu Rev Biochem.

[69] Banath JP, Banuelos CA, Klokov D, MacPhail SM, Lansdorp PM, Olive PL (2009). Explanation for excessive DNA single-strand breaks and endogenous repair foci in pluripotent mouse embryonic stem cells. Exp Cell Res.

[70] Zhao B, Zhang WD, Duan YL, Lu YQ, Cun YX, Li CH (2015). Filia is an ESC-specific regulator of DNA damage response and safeguards genomic stability. Cell Stem Cell.

[71] Xiong J, Todorova D, Su NY, Kim J, Lee PJ, Shen Z (2015). Stemness factor Sall4 is required for DNA damage response in embryonic stem cells. J Cell Biol.

[72] Zalzman M, Falco G, Sharova LV, Nishiyama A, Thomas M, Lee SL (2010). Zscan4 regulates telomere elongation and genomic stability in ES cells. Nature..

[73] Zhao Y, Yin X, Qin H, Zhu F, Liu H, Yang W (2008). Two supporting factors greatly improve the efficiency of human iPSC generation. Cell Stem Cell.

[74] Hong H, Takahashi K, Ichisaka T, Aoi T, Kanagawa O, Nakagawa M (2009). Suppression of induced pluripotent stem cell generation by the p53-p21 pathway. Nature..

[75] Kawamura T, Suzuki J, Wang YV, Menendez S, Morera LB, Raya A (2009). Linking the p53 tumour suppressor pathway to somatic cell reprogramming. Nature..

[76] Li H, Collado M, Villasante A, Strati K, Ortega S, Canamero M (2009). The Ink4/Arf locus is a barrier for iPS cell reprogramming. Nature..

[77] Marion RM, Strati K, Li H, Murga M, Blanco R, Ortega S (2009). A p53-mediated DNA damage response limits reprogramming to ensure iPS cell genomic integrity. Nature..

[78] Hussein SM, Batada NN, Vuoristo S, Ching RW, Autio R, Narva E (2011). Copy number variation and selection during reprogramming to pluripotency. Nature..

[79] Laurent LC, Ulitsky I, Slavin I, Tran H, Schork A, Morey R (2011). Dynamic changes in the copy number of pluripotency and cell proliferation genes in human ESCs and iPSCs during reprogramming and time in culture. Cell Stem Cell.

[80] Gore A, Li Z, Fung HL, Young JE, Agarwal S, Antosiewicz-Bourget J (2011). Somatic coding mutations in human induced pluripotent stem cells. Nature..

[81] Fu X, Xu Y (2012). Challenges to the clinical application of pluripotent stem cells: Towards genomic and functional stability. Genome Med.

