# Supplementary material for: BNIP3-dependent mitophagy safeguards ESC genomic integrity via preventing oxidative stress-induced DNA damage and protecting homologous recombination
Source: Cell Death Dis. 2022 Nov 19;13(11):976. doi: 10.1038/s41419-022-05413-4 (PMC9675825; doi:10.1038/s41419-022-05413-4)
Supplement: Supplementary file 1 — Supplemental Figure legends [file 41419_2022_5413_MOESM1_ESM.docx]

**Supplementary Figure 1.** BNIP3 maintains mouse ESC mitochondrial homeostasis. (**A**) Deletion of Bnip3 does not induce ESC apoptosis. *Bnip3^+/+^* and *binp3^-/-^* ESCs were stained with PI and Annexin V and analyzed by a FACS. (**B**) PI and Annexin V double-negative ESCs (shown in A) were counted as viable cells. Data shown as mean ± SD, n=3; NS, no significant difference. (**C**) Electron microscopy reveals *Bnip3^+/+^* and *bnip3^-/-^ ESC* morphology. (**D**) Evaluation of mitophagy in bnip3^-/-^ ESCs and rescued ESCs with Mito-Keima. ESCs with Mito-Keima expression either under normal conditions or FCCP treatment (10 nM for 3 h) were analyzed with a confocal laser scanning microscope for Mito-Keima imaging (Excitation: 440nm and 590nm for mitochondria in neutral and acidic pH environments, respectively).

**Supplementary Figure 2**. NAC treatments inhibited the activation of ATM and p53 in *bnip3^-/-^* ESCs. (**A**) Quantifications of each protein expression in (**Figure 2 A**). Data are shown as mean ± SD, n=3; ***, P<0.001; Student’s t-test. (**B**) Quantifications of each protein expression in (**Figure 2 E**). Data are shown as mean ± SD, n=3; **, P<0.01; ***, P<0.001, Student’s t-test. (**C**) Quantifications of each protein expression in (**Figure 2 G**). Data are shown as mean ± SD, n=3; *, P<0.05; **, P<0.01; ***, P<0.001; Student’s t-test. (**D**) Activation of ATM and p53 in bnip3^-/-^ ESCs was suppressed by NAC treatment. Western blot detection of p-ATM, ATM, p-p53 and p53 in Bnip3^+/+^ ESCs and Bnip3^-/-^ ESCs with or without NAC treatment. Actin served as the loading control.

**Supplementary Figure 3.** Generation of Bnip3 & ATM KO and Bnip3 & p53 KO ESCs by CRISPR-Cas9 system. (**A**) Schematic of Cas/sgRNA-targeting sites in ATM genome loci. (**B**) Western blot to identify the knockout of ATM. (**C**) Schematic of Cas/sgRNA-targeting sites in p53 genome loci. (**D**) Western blot to identify the knockout of p53.

**Supplementary Figure 4**. BNIP3-mediated mitophagy maintains ESC identity by preventing p53-dependent differentiation. (**A**) Quantifications of each protein expression in (**Figure 2 H**). Data are shown as mean ± SD, n=3; *, P<0.05; **, P<0.01; ***, P<0.001; Student’s t-test. (**B**) BNIP3 maintains ESC identity by preventing p53-dependent differentiation. AP staining of *Bnip3^+/+^*, *bnip3^-/-^* and *bnip3^-/-^* & *p53^-/-^* ESCs. (**C**) Real-time PCR was used for detecting the pluripotent gene expression in *Bnip3^+/+^*, *bnip3^-/-^* and *bnip3^-/-^* & *p53^-/-^* ESCs. The data are shown as mean ± SD, n=3; **, P<0.01; ***, P<0.001; Student’s t-test.

**Supplementary Figure 5.** Excessive activation of AMPK deteriorates the homologous recombination. (**A**) Deletion of Bnip3 decreases ATP production by mitochondrial respiration in ESCs. Data are shown as mean ± SD, n=3; *, P<0.05; **, P<0.01; Student’s t-test. (**B**) Quantifications of indicated protein expression in (**Figure 3 D**). Data are shown as mean ± SD, n=3; ***, P<0.001; Student’s t-test. (**C**) Quantifications of indicated protein expression in (**Figure 3 E**). Data are shown as mean ± SD, n=3; ***, P<0.001; Student’s t-test. (**D**) Excessive AMPK activation leads to decreased RAD51 expression. Western blot detection of p-AMPK, AMPK, γH2AX and RAD51 in Bnip3^+/+^ESCs (Mock, AICAR or Oligomycin treated) and *bnip3^-/-^* ESCs. Actin served as the loading control. (**E**) Western blot detection of γH2AX and RAD51 in Bnip3^+/+^ and bnip3^-/-^ ESCs with indicated treatments. (**F**) Quantifications of each protein expression in (**E**). Data are shown as mean ± SD, n=3; *, P<0.05; **, P<0.01; ***, P<0.001; NS, no significant difference, Student’s t-test.

**Supplementary Figure 6.** Bnip3 overexpression decreases ROS production and safeguards mitochondrial function in iPSCs. (**A**) Cells with SSEA-1 expression at reprogramming day 3. (**B**) Bnip3 overexpression decreases ROS production during reprogramming. Reprogramming cells with SSEA-1 expression in (**A**) were stained with DCFH-DA. (**C**) Bnip3 overexpression decrease ROS production in established iPSCs. (**D**) Bnip3 overexpression decrease ROS generation. We stained iPSCs-Vector and iPSCs-Bnip3 with DCFH-DA, and analyzed by a FACS. Data are shown as mean ± SD, n=3; **, P<0.01; Student’s t-test. (**E**) Oxygen consumption rates (OCRs) of iPSCs-Vector and iPSCs-Bnip3. (**F**) Extracellular acidification rate (ECAR) of iPSCs-Vector and iPSCs-Bnip3. (**G**) Bnip3 overexpression enhance ATP production by both glycolysis and mitochondrial respiration in iPSCs. Data are shown as mean ± SD, n=3; *, P<0.05; ***, P<0.001; Student’s t-test.
