# Supplementary figures and images for: BNIP3-dependent mitophagy safeguards ESC genomic integrity via preventing oxidative stress-induced DNA damage and protecting homologous recombination
Source: Cell Death Dis. 2022 Nov 19;13(11):976. doi: 10.1038/s41419-022-05413-4 (PMC9675825; doi:10.1038/s41419-022-05413-4)

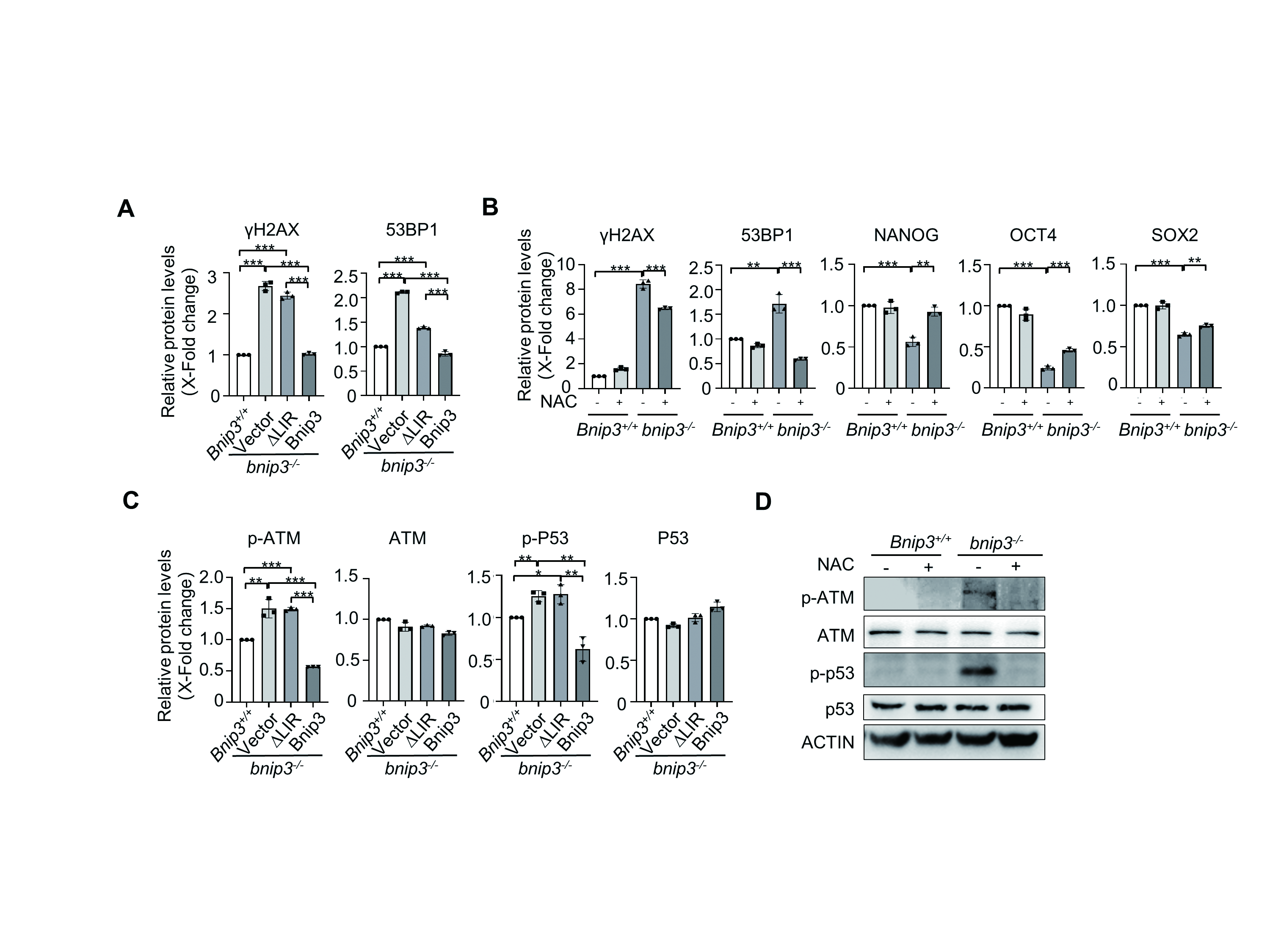

Supplement: Supplementary file 3 — Supplementary Figure 2 [file 41419_2022_5413_MOESM3_ESM.tif]

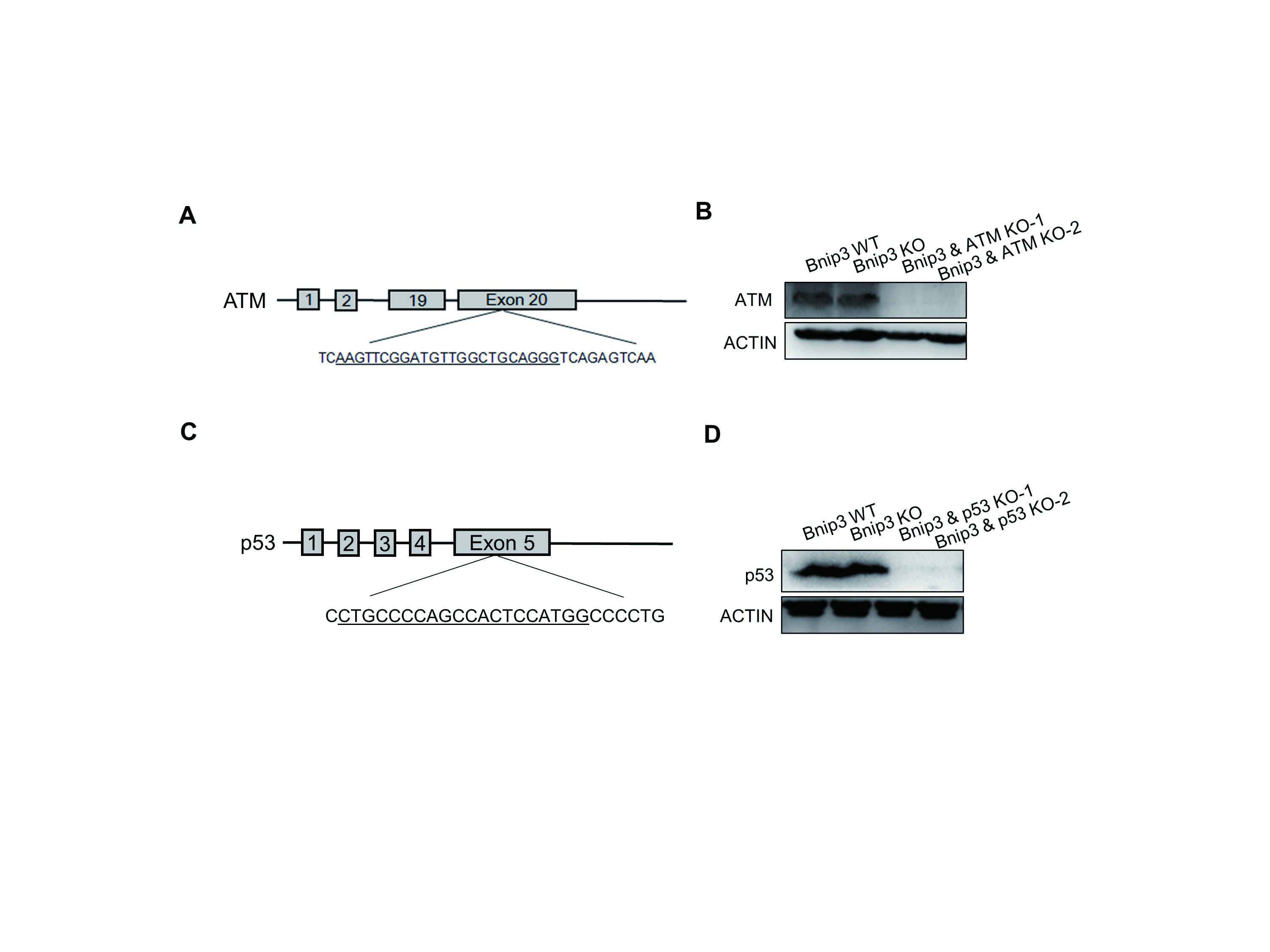

Supplement: Supplementary file 4 — Supplementary Figure 3 [file 41419_2022_5413_MOESM4_ESM.tif]

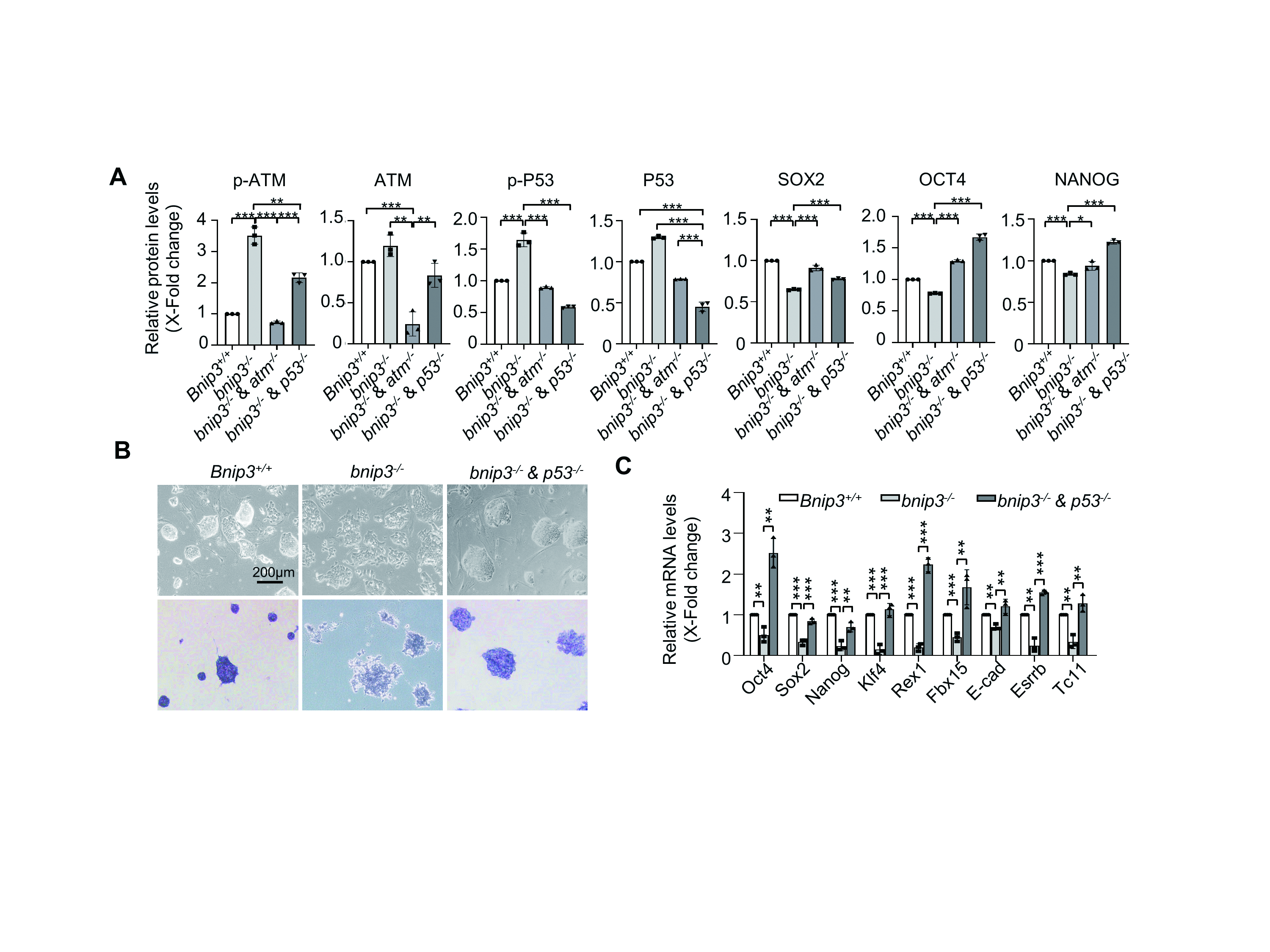

Supplement: Supplementary file 5 — Supplementary Figure 4 [file 41419_2022_5413_MOESM5_ESM.tif]

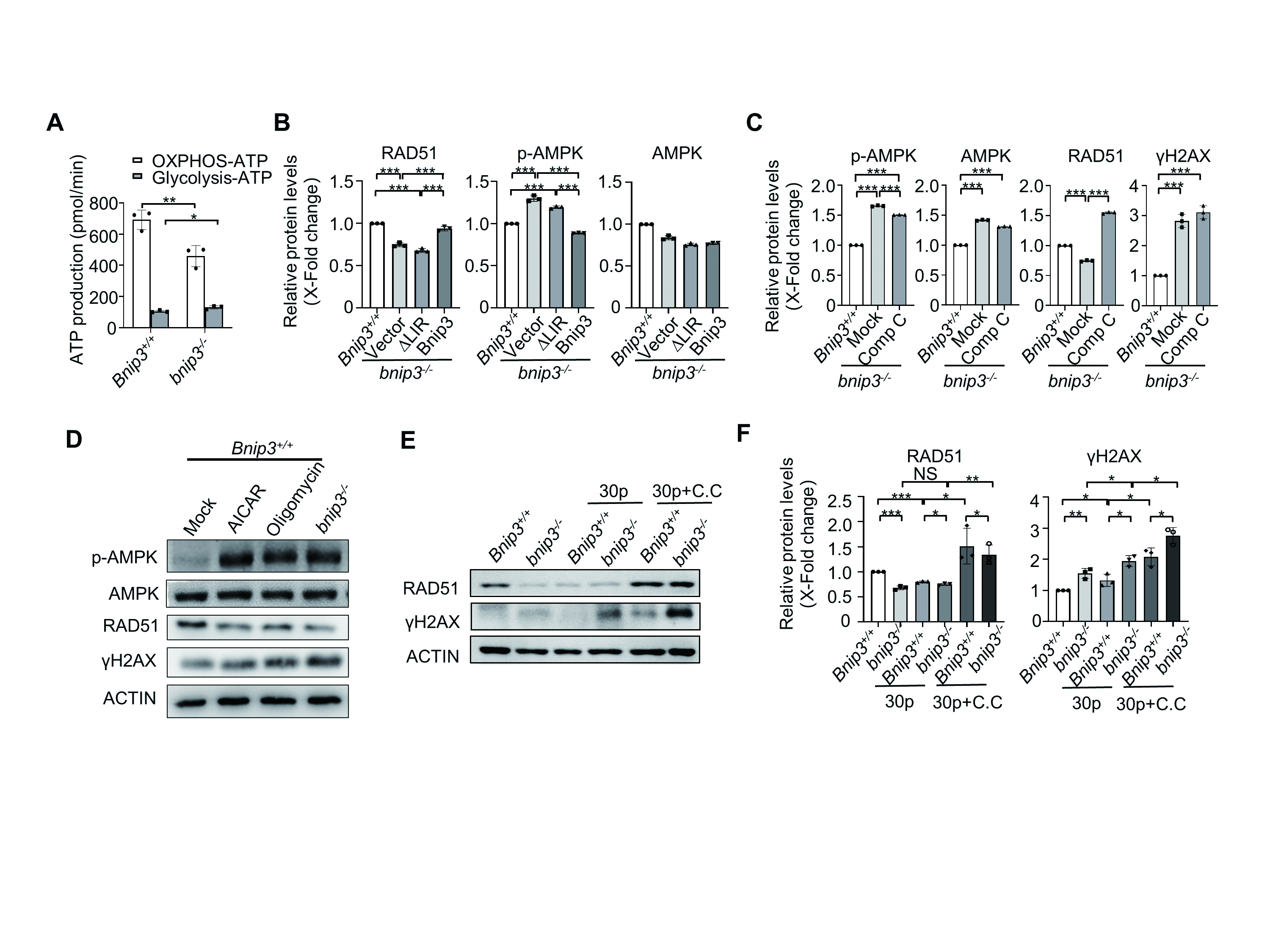

Supplement: Supplementary file 6 — Supplementary Figure 5 [file 41419_2022_5413_MOESM6_ESM.tif]

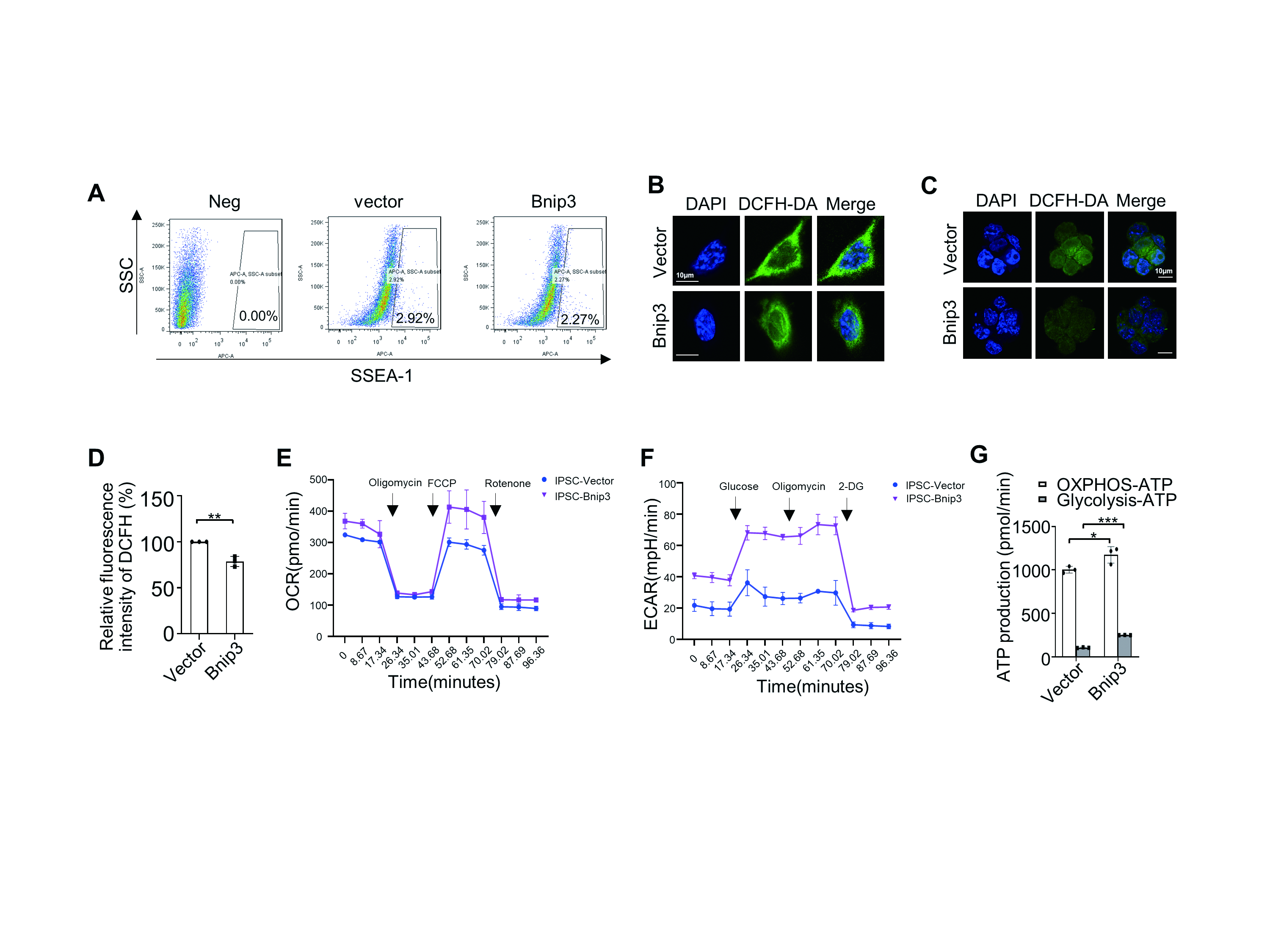

Supplement: Supplementary file 7 — Supplementary Figure 6 [file 41419_2022_5413_MOESM7_ESM.tif]
