# Supplementary material for: BNIP3-dependent mitophagy safeguards ESC genomic integrity via preventing oxidative stress-induced DNA damage and protecting homologous recombination
Source: Cell Death Dis. 2022 Nov 19;13(11):976. doi: 10.1038/s41419-022-05413-4 (PMC9675825; doi:10.1038/s41419-022-05413-4)
Supplement: Supplementary file 8 — Original Data File [file 41419_2022_5413_MOESM8_ESM.pptx]

## Slide 1
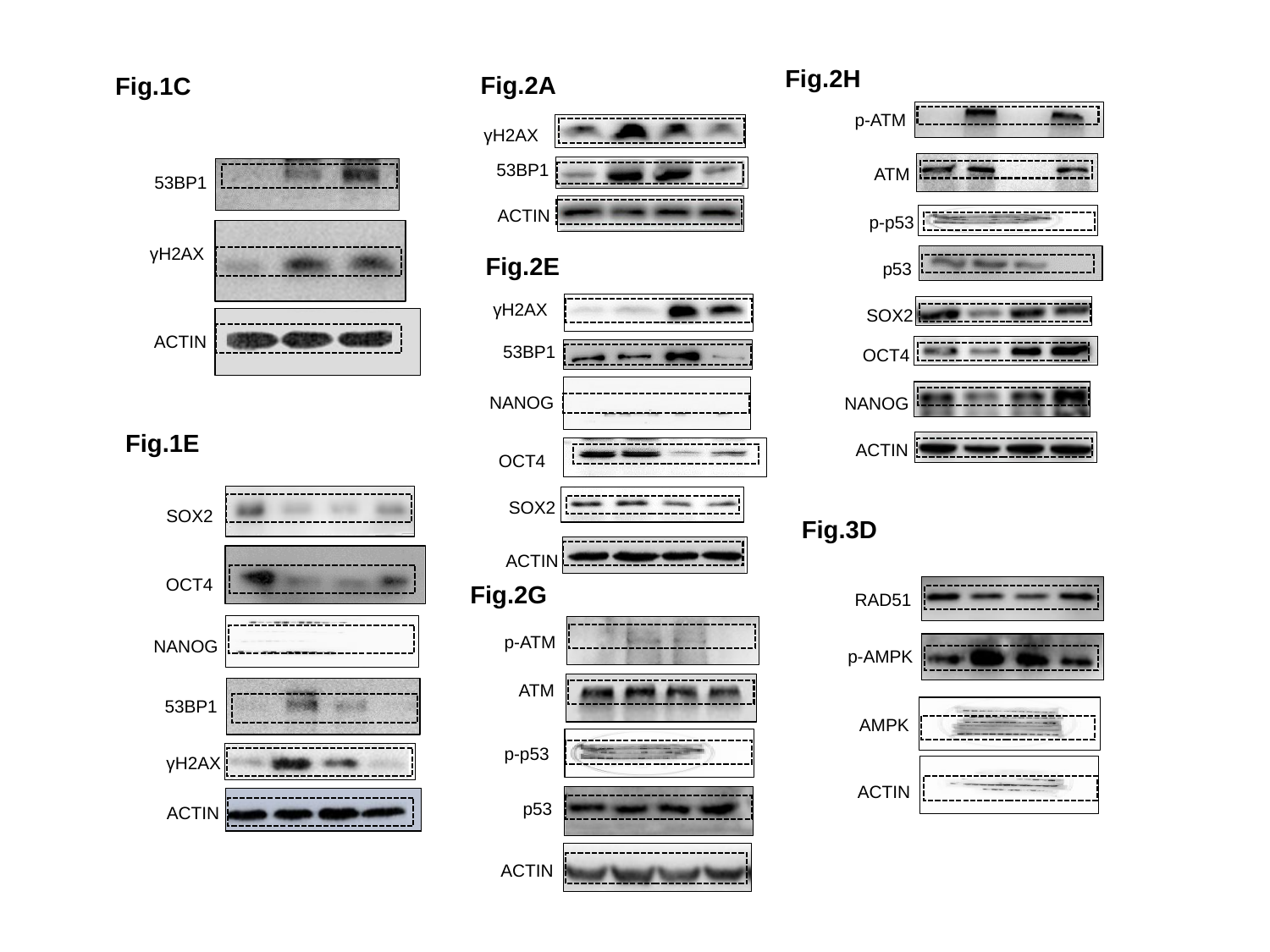

Fig.2H
Fig.2A
Fig.1C
p-ATM
ATM
p-p53
p53
SOX2
OCT4
NANOG
ACTIN
γH2AX
53BP1
ACTIN
53BP1
γH2AX
Fig.2E
γH2AX
53BP1
NANOG
OCT4
SOX2
ACTIN
ACTIN
Fig.1E
SOX2
OCT4
NANOG
53BP1
γH2AX
ACTIN
Fig.3D
Fig.2G
RAD51
p-AMPK
AMPK
ACTIN
p-ATM
ATM
p-p53
p53
ACTIN

## Slide 2
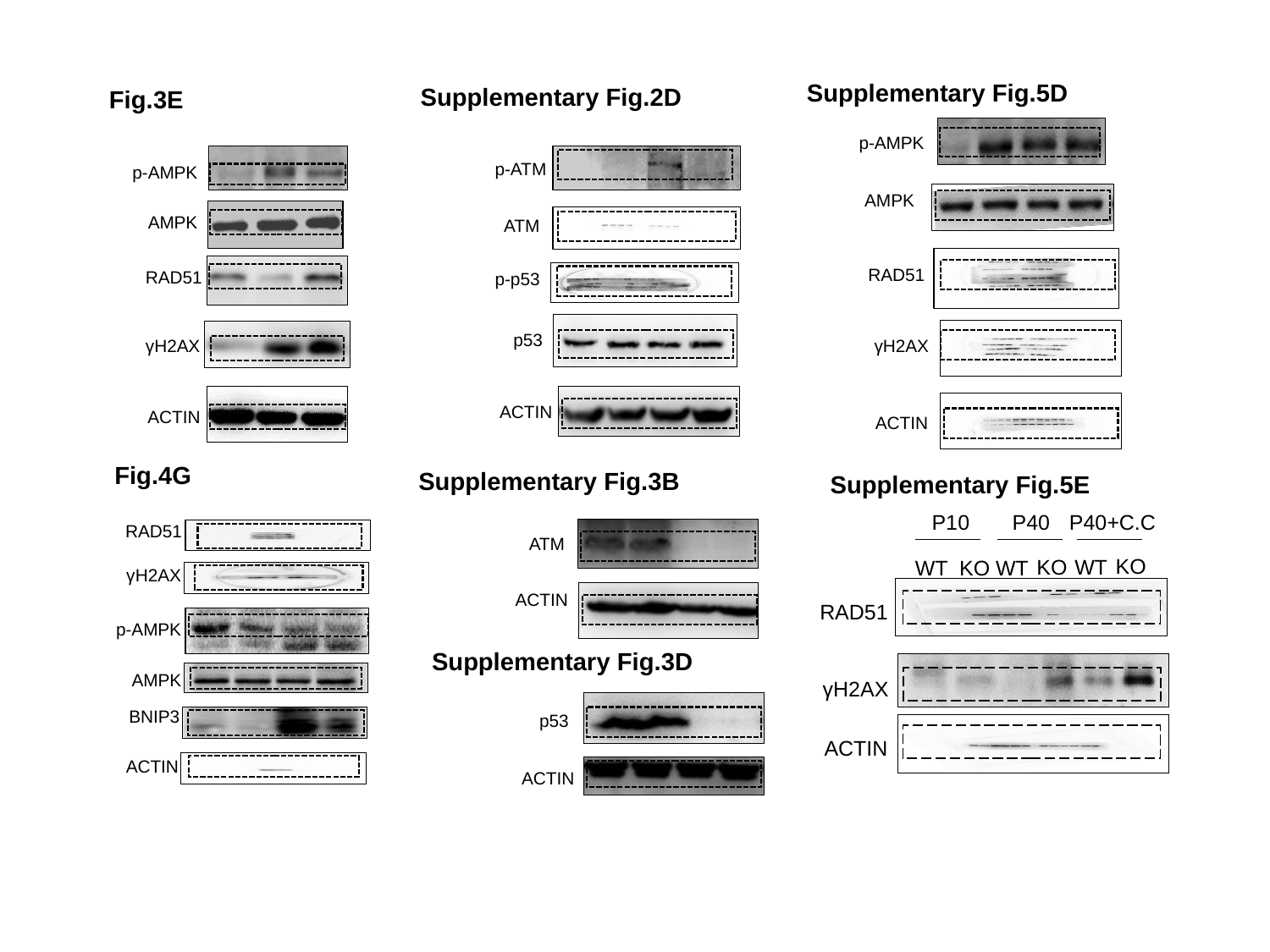

Supplementary Fig.5D
Supplementary Fig.2D
Fig.3E
p-AMPK
AMPK
RAD51
γH2AX
ACTIN
p-AMPK
AMPK
RAD51
γH2AX
ACTIN
p-ATM
ATM
p-p53
p53
ACTIN
Fig.4G
Supplementary Fig.3B
Supplementary Fig.5E
P40+C.C
P10
P40
KO
KO
WT
WT
KO
WT
RAD51
γH2AX
ACTIN
RAD51
γH2AX
p-AMPK
AMPK
BNIP3
ACTIN
ATM
ACTIN
Supplementary Fig.3D
p53
ACTIN
